# Onecut Regulates Core Components of the Molecular Machinery for Neurotransmission in Photoreceptor Differentiation

**DOI:** 10.3389/fcell.2021.602450

**Published:** 2021-03-18

**Authors:** Quirino Attilio Vassalli, Chiara Colantuono, Valeria Nittoli, Anna Ferraioli, Giulia Fasano, Federica Berruto, Maria Luisa Chiusano, Robert Neil Kelsh, Paolo Sordino, Annamaria Locascio

**Affiliations:** ^1^Department of Biology and Evolution of Marine Organisms, Stazione Zoologica Anton Dohrn, Naples, Italy; ^2^Department of Research Infrastructures for Marine Biological Resources, Stazione Zoologica Anton Dohrn, Naples, Italy; ^3^Department of Agriculture, Università degli Studi di Napoli Federico II, Portici, Italy; ^4^Department of Biology and Biochemistry and Centre for Regenerative Medicine, University of Bath, London, United Kingdom

**Keywords:** transcriptomic analysis, ascidian, eye, ocellus, transcription factor, genetic pathway

## Abstract

Photoreceptor cells (PRC) are neurons highly specialized for sensing light stimuli and have considerably diversified during evolution. The genetic mechanisms that underlie photoreceptor differentiation and accompanied the progressive increase in complexity and diversification of this sensory cell type are a matter of great interest in the field. A role of the homeodomain transcription factor Onecut (Oc) in photoreceptor cell formation is proposed throughout multicellular organisms. However, knowledge of the identity of the Oc downstream-acting factors that mediate specific tasks in the differentiation of the PRC remains limited. Here, we used transgenic perturbation of the *Ciona robusta* Oc protein to show its requirement for ciliary PRC differentiation. Then, transcriptome profiling between the trans-activation and trans-repression Oc phenotypes identified differentially expressed genes that are enriched in exocytosis, calcium homeostasis, and neurotransmission. Finally, comparison of RNA-Seq datasets in *Ciona* and mouse identifies a set of Oc downstream genes conserved between tunicates and vertebrates. The transcription factor Oc emerges as a key regulator of neurotransmission in retinal cell types.

## Introduction

The vertebrate retina is an excellent model tissue to investigate the developmental programs underlying nervous system complexity. During evolution, photoreceptor cells (PRC) have been organized in many different manners in order to enable organisms to perceive light and recognize light direction and, in vertebrates, form high-resolution imaging (Lamb, 2009). PRC convert light into nerve impulses and then transmit those impulses to the brain. Their development and maintenance require finely tuned gene expression and morphogenetic mechanisms (Hennig et al., 2008). Besides the fact that considerable progress in understanding the genetic network that controls PRC differentiation has been made over the last 20 years, our comprehension still contains many gaps. The *Onecut* (*Oc*) gene family of transcription factor coding genes plays key roles in the development of the vertebrate eye (Kropp and Gannon, 2016). The *Oc* gene was identified in invertebrates (*Drosophila melanogaster, Caenorhabditis elegans, Strongylocentrotus purpuratus*) for its expression during neurogenesis (Cassata et al., 1998; Lannoy et al., 1998; Nguyen et al., 2000; Otim et al., 2004). In *Drosophila, D-Onecut* positively regulates the expression of the *rhodopsin1* gene during late differentiation of PRC (Nguyen et al., 2000). In a previous study, we identified the ascidian *Oc* ortholog as a positive regulator of the retinal homeobox gene *Rx*, an essential factor in vertebrate eye development (D'Aniello et al., 2011). In ascidian tunicates, the closest relatives of vertebrates, *Oc* functions in neural cell type and PRC differentiation (Sasakura and Makabe, 2001; D'Aniello et al., 2011; Pezzotti et al., 2014).

In vertebrates, *Oc* genes have maintained neurogenic roles described in non-vertebrates, evolving novel functions according to the increased complexity of the nervous system (reviewed in Kropp and Gannon, 2016). In mouse spinal cord, *Oc* genes are required for proper diversification of specific motor neurons and interneurons through direct regulation of *Lmx1a* and *Isl1/2* gene expression in a network with *Neurog2, Nkx6.1*, and *Pax6* (Chakrabarty et al., 2012; Kim et al., 2015; Kabayiza et al., 2017; Harris et al., 2019). The lack of Oc proteins leads to multiple defects in both central and peripheral nervous systems, including atrophy of motor neurons, failure of neuromuscular junctions and Renshaw cell interneurons to form properly, defective reorganization of cerebellar Purkinje cells, and reduction of midbrain dopaminergic neurons (Audouard et al., 2012, 2013; Stam et al., 2012; Yuan et al., 2015). A role of Oc homeodomain proteins in differential chromatin remodeling and accessibility has recently been proposed (Velasco et al., 2017; van der Raadt et al., 2019). Despite a significant amount of data from non-vertebrate and vertebrate models on *Oc* gene role in neural development, little information is available on the genetic cascade regulated by Oc homeodomain proteins in PRC formation (Nguyen et al., 2000).

Oc factors exert different roles during the formation of various territories of the vertebrate visual system (Wu et al., 2012, 2013; Emerson et al., 2013; Goetz et al., 2014; Klimova et al., 2015; Madelaine et al., 2017). Target mRNA degradation of three zebrafish *oc* genes (*oc1, oc2*, and *oc-like*) by miR-9 binding is essential for eye angiogenesis (Madelaine et al., 2017). A role of Oc1 and Oc2 downstream of Pax6 as regulators of *Lim1* and *Prox1* in the differentiation and maintenance of horizontal cells (HC), retinal ganglion cells (RGC), PRC, and amacrine cells (AC) was evidenced by loss-of-function studies in mice (Wu et al., 2012, 2013; Sapkota et al., 2014; Klimova et al., 2015). In particular, the two murine Oc factors regulate PRC migration to the boundary of the neuroblast layer and selectively promote cone differentiation from PRC precursors while repressing rod fate (Wu et al., 2012; Emerson et al., 2013; Goetz et al., 2014; Sapkota et al., 2014; Jean-Charles et al., 2018).

Besides progress in understanding the biological significance and the mechanisms of action of *Oc* genes in vertebrate eye formation, how gene functions evolved in chordates remain poorly defined. Oc roles have been difficult to define in vertebrates owing to the presence of multiple gene copies with redundant functions. Downregulation of both mouse *Oc1* and *Oc2* results in a more severe retinal phenotype (Goetz et al., 2014; Sapkota et al., 2014). Ascidians occupy a key phylogenetic position for unveiling unique and common features of the *Oc* genetic cascade in chordates. Ascidian PRC are ciliary vertebrate-type cells that control two distinct visuomotor pathways with a dynamic series of responses, ranging from a looming-object escape behavior to a negative phototaxis for directional swimming (Salas et al., 2018). Using transactivating and repressing forms of the Onecut homeodomain and differential transcriptomics, we identify new downstream targets of Oc that operate in synaptic signaling of PRC in the model ascidian *Ciona robusta*. In addition, our comparative analysis of *Ciona* and mouse Oc-depleted transcriptomes offers novel insights into the specific transcriptional pathways and functions mediated by the *Oc* genetic pathway in PRC differentiation before the origin of vertebrates.

## Materials and Methods

### Animals

Adult specimens of *Ciona* were collected in the Gulf of Taranto, Italy, by hand picking at low depth and transported in seawater tanks to the facilities of Stazione Zoologica Anton Dohrn (SZN). Animals were acclimatized at ~20°C for 2–3 days in open system tanks and fed every day with a solution of marine microalgae concentrates (Shellfish Diet 1800™ Instant Algae®). Subsequently, they were exposed to continuous lighting for a few days in order to accumulate mature gametes and to prevent gamete spawning.

### Constructs and Transgenesis

**Figure 1:**
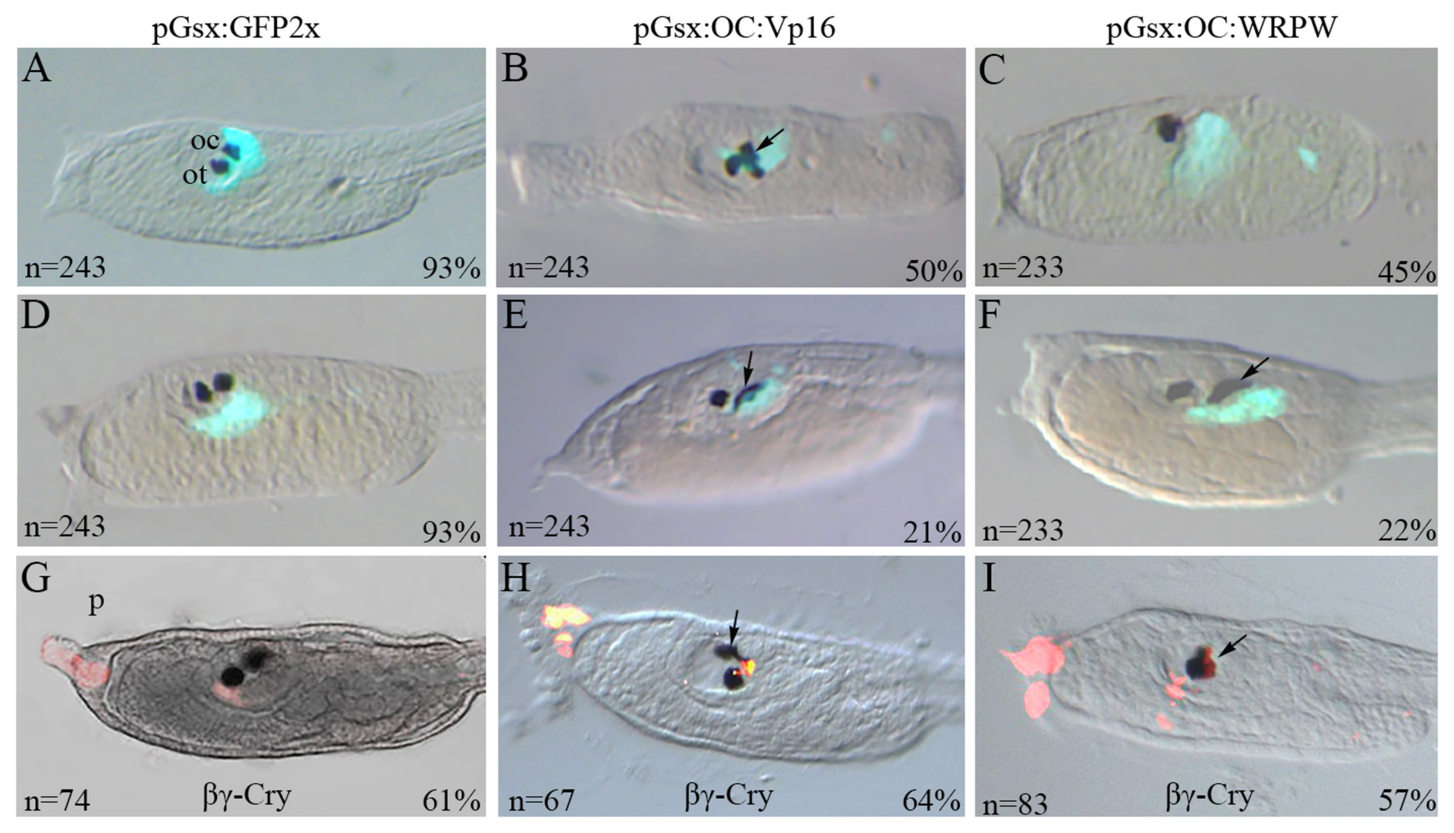
Transgenic manipulation of *Ciona Onecut* during ocellus formation. Typical phenotypes of stage 26 larvae co-electroporated with **(A,D)** the pGsx>GFP2x control construct and **(B,C)** the active form pGsx>OC::Vp16 (pGsx>OC::Act) or with **(E,F)** the repressive form pGsx>OC::WRPW (pGsx>OC::Rep). **(A,D)** Proportion of transgenic control larvae showing GFP expression and **(B,C,E,F)** percentages of altered phenotypes observed in electroporated larvae are indicated; *n*, number of embryos analyzed in each condition. The percentages refer to the altered phenotypes with respect to the total GFP-positive larvae. **(G–I)** Immunofluorescent assay with anti-βγ-crystalline antibody on larvae electroporated with the OC::Act **(H)** and OC::Rep **(I)** constructs. As control, the larvae electroporated with the pGsx>GFP2x construct **(G)**. *n*, number of embryos analyzed in each condition. Percentages indicate the number of embryos with the corresponding phenotype in each condition as described in Supplementary Table 5. *n*, number of embryos analyzed in each condition. In aquamarine green, the Gsx>GFP signal was used as internal control **(A–F)**. In red is the expression of βγ-crystalline **(G–I)**. The images were obtained with confocal microscopy. For all embryos, the anterior is on the left, lateral view.

The pGsx>OC::Act and pGsx>OC::Rep constructs were prepared by cloning in a pBSII backbone the Gsx promoter (Esposito, 2014; Palladino, 2017; Hudson et al., 2019) and the *Ciona* OC full-length coding sequence fused in frame with the Vp16 activator or WRPW repressor domains (D'Aniello et al., 2011). *C. robusta* transgenic embryos were obtained *via* electroporation as previously described (Locascio et al., 1999). In all electroporation experiments, the pGsx>GFP2x construct was used as internal control for the selection of GFP-positive electroporated embryos. About 200–300 embryos were electroporated in each experiment and incubated at 18°C until they reached the desired developmental stages. The developmental stages were established according to the FABA ascidian database (Hotta et al., 2007; https://www.bpni.bio.keio.ac.jp/chordate/faba/1.4/top.html). Each electroporation experiment was repeated at least 10 times and GFP-positive embryos (corresponding to 80–90% of the developed ones) were used partly for statistical analyses and partly were collected and treated for subsequent experiments [e.g., whole-mount *in situ* hybridization (WISH), immunostaining, cells sorting, and RNA extraction]. For phenotypic analyses of transgenic larvae, about 80 GFP-positive larvae (stage 26), in three experimental replicates, were analyzed under a light microscope and evaluated for their phenotypic alterations of pigmented sensory organs. A total of 233 and 243 transgenic larvae were counted and used for the percentage calculation of the more representative phenotypes observed in the OC::Act and OC::Rep conditions (Supplementary Table 5; Figure 1). About 40 GFP-positive transgenic embryos for each condition were used in every WISH or immunostaining experiment, made in biological duplicate/triplicate. The raw data and percentages of the altered gene expression levels observed in the various experimental replicates are shown in Supplementary Table 5. A Zeiss Axio Imager M1 microscope equipped with an Axiocam digital camera was used for image capture of a selection of transgenic embryos. Raw images of selected transgenic larvae and embryos have been deposited in the ANISEED database and can be viewed at the following link: http://dev.aniseed.cnrs.fr/aniseed/experiment/list_insitus_by_pub?pub_id=321. Pictures were edited with Adobe Photoshop CS5 and adjustments, where applied, were only for clarity without affecting any essential part of the image.

### mRNA Sequencing

Cell dissociation and magnetic-activated cell sorting (MACS) were performed as described (Wang et al., 2018). The pGsx>cd8::GFP construct was prepared by replacing pMesp promoter with pGsx in pMesp>cd8::GFP. The pMesp>cd8::GFP and the pMyoD>cd4::mCherry constructs were a generous gift of Dr. L. Christiaen (New York University). Total RNA extraction from sorted samples was performed using the RNAqueous-micro kit (Ambion, #1931). The libraries were prepared from 10 ng of purified RNA with SMART-Seq Low Input RNA Kit (Clontech Laboratories, Inc.) according to the manufacturer's instructions. Libraries were quantified using the Tape Station 4200 (Agilent Technologies). The pooled samples were subject to cluster generation and sequencing using an Illumina HiSeq2500 System by the Genomix4Life s.r.l. (https://www.genomix4life.com). The mapping of paired-end reads was performed using the bioinformatics tool STAR (version 2.5.0a) (Dobin et al., 2012). The quantification of transcripts expressed for each of the three replicates of the sequenced samples was performed using HTSeq-count (Anders et al., 2014). The raw sequence files generated (fastq files) underwent quality control analysis using FastQC (http://www.bioinformatics.babraham.ac.uk/projects/fastqc/). The reference genome version was the assembly Ciona_intestinalis.KH.78 (https://www.ensembl.org/Ciona_intestinalis/Info/Index). All the Ensembl gene name codes were shortened for simplicity (e.g., ENS_07757 for ENSCING00000007757). Genes were considered differentially expressed (DEGs) when count filtered for padj <0.05 and FC >|1.5|.

SRA records are accessible with the following link: https://www.ncbi.nlm.nih.gov/sra/PRJNA680353.

### Gene Orthology Analysis

To confirm or update the function associated to *Ciona* gene annotations (available at https://www.ensembl.org/Ciona_intestinalis/Info/Index), transcript sequences were aligned vs. the UniProt Swiss-Prot database (version of November 2016) using BLASTx (version 2.2.29+, e-value: 0.001) (Camacho et al., 2009). The Best BLAST Hit function was considered to associate the most probable functionality to assign to each transcript structure. The DEGs showing no similarity based on the BLASTx vs. UniProt Swiss-Prot database (at https://www.uniprot.org/uniprot/?query=reviewed:yes, version of November 2016) were also blasted (e-value: 0.001) vs. the UniProt TrEMBL database (at https://www.uniprot.org/uniprot/?query=reviewed:no version of November 2016).

For the DEGs showing no results vs. UniProt, we also performed the online version of the BLASTx (https://blast.ncbi.nlm.nih.gov/Blast.cgi) vs. the “non-redundant” (nr) protein database of NCBI, and then we searched for domains using INTERPRO (https://www.ebi.ac.uk/interpro/) (Table 1).

**Table 1:** Genes differentially expressed in both OC::Act and OC::Rep embryos.

**Ensembl code simplified**	**Ensembl/NCBI description**	**OC::Act log2FC**	**OC::Rep log2FC**	**UniProt ID**	**UniProt protein description**
ENS_05771	NA	2.31	−1.79	F6RT14	Uncharacterized protein
ENS_04964	NA	1.95	−1.01	F6X9N9	Uncharacterized protein
ENS_07348	GTP-binding protein Di-Ras2-like (Diras)	1.93	−0.93	Q5PR73	GTP-binding protein Di-Ras2
ENS_22831	NA	1.80	−1.06	H2XT45	Uncharacterized protein
ENS_05712	Neuroendocrine convertase 2-like (Pcsk2)	1.69	−1.11	P28841	Neuroendocrine convertase 2
ENS_02864	Ras-related protein RABA2b-like (Rab11a)	1.61	−1.11	O04486	Ras-related protein RABA2a
ENS_05701	Secretagogin-like (Scgn-like)	1.44	−1.04	F6WQG5	Uncharacterized protein
ENS_22421	NA	1.42	−1.33	H2XRK0	Uncharacterized protein
ENS_06770	Twitchin-like (Tmtc2)	1.29	−0.89	Q6DCD5	Transmembrane and TPR repeat-containing protein 2
ENS_06816	Synaptotagmin (Syt)	1.20	−0.87	Q5R4J5	Synaptotagmin-1
ENS_20046	Complexin-2 (Cplx2)	1.19	−1.00	P84087	Complexin-2
ENS_19724	Probable cationic amino acid transporter (Slc7a14)	1.05	−1.10	Q8BXR1	Probable cationic amino acid transporter
ENS_03816	NA	1.04	−0.99	F6ZT94	Uncharacterized protein
ENS_23426	NA	1.16	−1.42	H2XJT7	Uncharacterized protein
ENS_21806	Sodium-dependent serotonin transporter-like (Slc6)	−1.48	−2.08	H2XWG9	Uncharacterized protein
ENS_23151	NA	−2.06	−1.15	H2XRW0	Uncharacterized protein
ENS_07757	Sodium-dependent serotonin transporter-like (Slc6a2)	−1.36	−1.74	O35899	Sodium-dependent serotonin transporter
ENS_20001	FK506-binding protein 3 (FKBP25/3)	−1.34	−0.88	H2XP73	Uncharacterized protein
ENS_14534	14-Alpha-glucan-branching enzyme-like (Gbe1)	−1.27	−1.11	Q08047	1,4-Alpha-glucan-branching enzyme 2, chloroplastic/amyloplastic

*For each gene, the simplified Ensembl code, the Ensembl/NCBI description, the log2 fold change (log2FC) in OC::Act and OC::Rep conditions, and the UniProt ID description resulting from BLASTx are reported*.

### Comparative Transcriptomics

The orthologous genes in *Ciona* and mouse were identified performing (i) a tBLASTx (e-value: 0.001) of *Ciona* transcripts vs. mouse transcripts, (ii) a tBLASTx (e-value: 0.001) of mouse transcripts vs. *Ciona* transcripts, and (iii) the Transcriptologs approach (Ambrosino and Chiusano, 2017) in order to find the bidirectional best hits (BBHs). Orthologous and paralogous genes were organized in gene networks, according to Ambrosino et al. (2018).

### Whole-Mount *in situ* Hybridization

The following WISH probes were obtained from clones of the *Ciona* Ghost EST collection database (Release 1): *Opsin1* (CiGC28m24), *Arrestin* (CiGC28m09), *Pax3/7* (CiGC42e20), and *Synaptotagmin* (CiGC26m18). PCR amplification of the other transcription templates was performed with oligos shown in Supplementary Table 6. Single and double fluorescent WISH on *Ciona* embryos were performed as previously described by Pezzotti et al. (2014). Differential interference contrast microscopy images were acquired with a Zeiss Axio Imager M1 microscope equipped with an Axiocam digital camera. Confocal microscopy images were acquired with a Zeiss LSM 510 META Laser Scanning Confocal Microscope (Jena, Germany). Pictures were edited with Adobe Photoshop CS5, and adjustments, where applied, were only for clarity without affecting any essential part of the image. Raw images of selected stained embryos are deposited in the ANISEED web page: http://dev.aniseed.cnrs.fr/aniseed/experiment/list_insitus_by_pub?pub_id=321.

## Results

### Ascidian *Onecut* Functions in Ocellus Differentiation

Ascidians have two sets of PRC that are tightly associated with the ocellus photosensitive organ. Their fine structure and the projection of the outer segments into the cup-shaped pigment cell have been analyzed by confocal and electron microscopy (Horie et al., 2008; Salas et al., 2018; Kourakis et al., 2019). The promoter of the ParaHox *Gsx* gene directs specifically the reporter expression in both the a9.33 and A9.14 cell lineages proposed as PRC precursors (Esposito, 2014; Oonuma et al., 2016; Esposito et al., 2017; Palladino, 2017). As described in Supplementary Figure 1, *Gsx* expression precedes that of *Oc* in these cells. We overexpressed the *Oc* coding sequence fused in frame with the VP16 (constitutively active) and WRPW (constitutively repressive) domains under the control of the *Gsx* promoter, with the aim to investigate the phenotypic consequences on PRC precursor development. Following electroporation of the pGsx>GFP2x construct, control larvae displayed the wild-type head phenotype, in which two pigmented sensory organs are clearly distinguishable, i.e., the otolith, which is a gravity sensory organ located ventrally in the anterior region of the sensory vesicle, and the photosensing ocellus more dorsally, in the wall of the vesicle (Figures 1A,D). When the constitutively active construct pGsx>OC::Vp16 (OC::Act) was used, 71% of transgenic larvae showed abnormal pigmented organs (Figures 1B,E). In particular, 50% of larvae showed supernumerary pigmented cells with modified size and shape (Figure 1B), while 21% only carried dysmorphic pigmented cells (Figure 1E); the remaining 30% were similar to the control (Supplementary Table 5). Looking at the sensory pigment cells in *Ciona* larvae carrying the constitutively repressive form pGsx>OC::WRPW (OC::Rep), 45% of them showed only one apparently normal pigmented cell in the sensory vesicle (Figure 1C), 25% had one larger and morphologically abnormal sensory pigment cell (Figure 1F), and 33% were similar to control larvae (Supplementary Table 5).

To understand the identity of surplus and altered pigment cells, i.e., whether they are ocellus and/or otolith, we performed an immunofluorescent assay with an antibody against βγ-crystalline, a protein specifically expressed in otolith and palps (Shimeld et al., 2005) (Figures 1G–I). In OC::Act larvae, βγ-crystalline antibody labeled the anterior sensory pigment cell (i.e., otolith) as expected, and no immunofluorescence was detected in supernumerary pigment cells. In OC::Rep larvae, βγ-crystalline immunoreactivity was always located in the single pigmented cell of the otolith (see Supplementary Table 5 for experimental percentages). These results indicate that the altered phenotype does not affect the otolith. Furthermore, the supernumerary pigment cells observed in OC::Act larvae suggest a possible direct role of the active form of *Oc* in the induction of pigmentation. Consistent with this hypothesis, *Ciona Oc* is expressed in pigment cell lineage in wild-type embryos (Cao et al., 2019), and the human *OC2* gene directly activates the *Microphthalmia-associated transcription factor* (*MITF*), which is essential for melanocyte differentiation (Jacquemin et al., 2001). DAPI labeling of OC::Act transgenic larvae evidenced that fully separated supernumerary pigment spots belong to distinct cells. The same assumption could not be established in the case of single enlarged spots due to the pigment interfering with the fluorescent signal (data not shown). Since the pigment cell lineage in *Ciona* is fully separated from that of PRC and the *Gsx* promoter is not active in pigment precursors, the anomalies in pigment cells observed in OC::Rep larvae suggest that the *Oc* repressor form is able to indirectly affect the adjacent, but tightly connected, pigment cell of the ocellus (Figure 1I).

**Figure 2:**
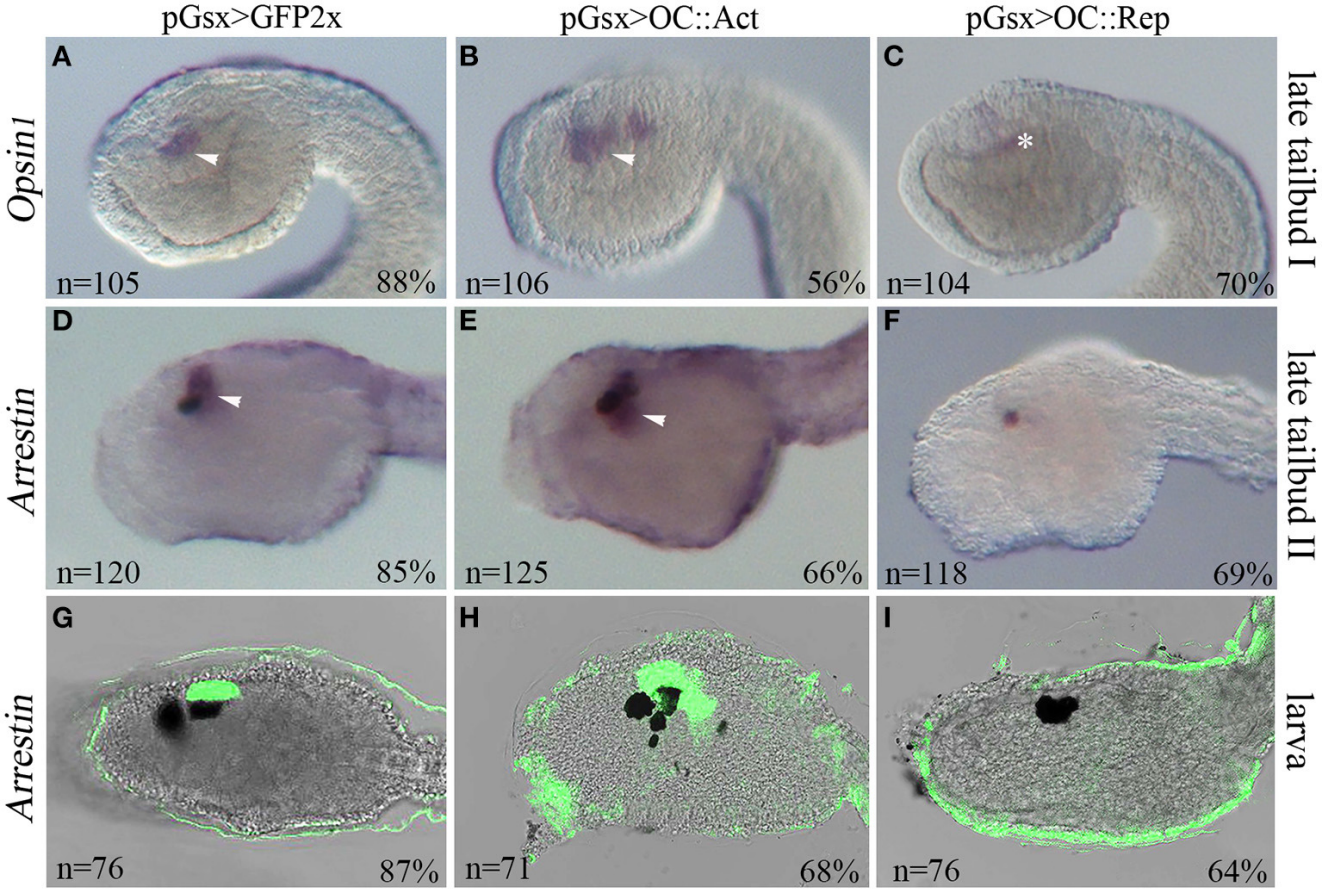
Manipulation of Oc activity interferes with photoreceptor development in *Ciona*. Expression of the photoreceptor cell (PRC) markers *Opsin1* and *Arrestin* in transgenic embryos at late tailbud I (stage 23) **(A–C)**, late tailbud II (stage 24) **(D–F)**, and larva (stage 26) **(G–I)**. Embryos electroporated with **(A,D)** pGsx>GFP2x control, **(B,E)** pGsx>OC::VP16 (pGsx>OC::Act), and **(C,F)** pGsx>OC::WRPW (pGsx>OC::Rep) constructs. **(A–C)** *Opsin1* whole-mount *in situ* hybridization (WISH). **(D–I)** *Arrestin* WISH. **(G–I)** Confocal merged images of *Arrestin* hybridized larvae. *n*, number of embryos analyzed in each condition. Percentages indicate the number of embryos with the corresponding expression profile in each condition as described in Supplementary Table 5. Arrowheads indicate the expression in PRC and their precursors, and asterisk indicates false-positive signal due to embryo shadowing. For all embryos, the anterior is on the left, lateral view.

In order to establish the type of ocellus alterations induced by *Oc* misexpression, we assayed two PRC marker genes *Opsin1* and *Arrestin*. Opsin1 is a visible light-sensitive G-protein-coupled receptor present in PRC of the larval ocellus, and it has the molecular properties of “an evolutionarily intermediate state between invertebrate-type and vertebrate visual opsins” (Kojima et al., 2017). Arrestin binds to light-activated phosphorylated rhodopsin and regulates the phototransduction in PRC (Horie, Orii and Nakagawa, 2005). They are co-expressed with *Oc* in differentiating PRC in the region of the sensory vesicle surrounding the ocellus pigment at the larval stage (Supplementary Figure 1) (Kusakabe et al., 2001; Nakagawa et al., 2002; Inada et al., 2003). In 56% of OC::Act tailbud embryos (stage 22/23), *Opsin1* expression in the sensory vesicle was markedly expanded (Figure 2B) in comparison with controls (Figure 2A). In contrast, markedly reduced or no expression of *Opsin1* was observed in 70% of OC::Rep embryos (Figure 2C). Likewise, expression of the *Arrestin* gene in the sensory vesicle was expanded in OC::Act transgenic embryos and completely or almost abolished in OC::Rep embryos at both the stage 24 (late tailbud II) and larva stage (Figures 2D–I), corresponding to the initial differentiation and complete differentiation phases of photoreceptor cells, respectively. Raw data and percentages of the results obtained on transgenic embryos are shown in Supplementary Table 5. In conclusion, we observed supernumerary PRC in OC::Act embryos and their loss in OC::Rep embryos. These results indicate the specificity of the phenotypic perturbation to the traits of the photosensitive ocellus.

### Transcriptomics Analysis of Transgenic Photoreceptor Cells in *Ciona*

The analysis of *Ciona* transgenic larvae suggested that the *Oc* gene is involved in PRC differentiation. In order to identify candidate effector genes associated with the *Oc* pathway, we employed a differential transcriptomic approach on PRC progenitor cells of *Ciona* OC::Act and OC::Rep transgenic embryos. After enrichment by MACS technology, neural precursors labeled by vectors containing the *Gsx* promoter were collected from late tailbud embryos (stage 23/24) and subjected to Illumina HiSeq2500 sequencing (Stranded RNA-Seq). We identified 9,967 *Ciona* genes whose expression was detected in the two transgenic conditions with at least one match with a protein present in the UniProt Swiss-Prot database. *Ciona* transgenic transcriptomes revealed about 500 genes with a significant change in expression level (DEGs), 469 of which were found in the OC::Act condition and 36 in the OC::Rep one (Supplementary Table 1, highlighted in yellow). Of note, 14 DEGs (log2 fold change > 2; *p*-value < 0.001) showed upregulation in OC::Act and downregulation in OC::Rep, lending themselves as candidate targets in the *Oc* genetic pathway implicated in PRC differentiation in *Ciona*. Other five DEGs were downregulated in both conditions (Table 1).

Genes showing opposite direction of change in transcriptional levels (Table 1, highlighted in orange) were predominant factors involved in synaptic transmission. The ENS_19724 (*Slc7a14*) gene encodes a serotonin transporter protein. In vertebrates, *Slc7a14* codes for a glycosylated, cationic amino acid transporter protein that has recently been found to underlie the autosomal recessive retinitis pigmentosa (Jin et al., 2014). Accordingly, *Slc7a14* loss-of-function phenotypes in mouse and zebrafish show structural and functional abnormalities in the retina (Jin et al., 2014; Zhuang et al., 2019). ENS_20046 (*Cplx2*) codes for a soluble protein that interacts with the SNARE complex, the molecular machinery of synaptic vesicle exocytosis (reviewed in Vaithianathan et al., 2015); mouse *Cplx2* is involved in synaptic neurotransmission of ON-bipolar neurons, those retinal cells that depolarize when stimulated by light (Reim et al., 2009; Landgraf et al., 2012; Vaithianathan et al., 2015). ENS_07348 (*Diras*) belongs to a small GTPase Ras superfamily that regulates many signaling pathways in neuronal functions, including neurotransmitter transmission, cell migration, neurite outgrowth, and neuron proliferation (Tada et al., 2012; Yeh and Hsu, 2016). In humans, *Diras3* is involved in functions and pathways relevant to glaucoma (Paylakhi et al., 2011). ENS_06770 (*Tmtc2*) encodes an endoplasmic reticulum tetratricopeptide repeat-containing adapter protein involved in calcium homeostasis that has been associated with primary open-angle glaucoma (Sunryd et al., 2014; Choquet et al., 2018; Graham et al., 2020). ENS_06816 (*Syt*) codes for a Ca^++^-sensor synaptotagmin protein that mediates the exocytotic fusion between vesicle and plasma membranes during synaptic transmission (Hui et al., 2009; Grassmeyer et al., 2019) and that regulates rat retina development *via* calcium binding to the C2AB domains (Chiang et al., 2012). Similarly, ENS_05712 (*Pcsk2*), ENS_05701 (*Scgn-like*), ENS_02864 (GTPase *Rab11a*), and ENS_20001 (*FKBP25/3*) genes encode for factors involved in different steps of neurotransmission as described in Supplementary Table 2. Interestingly, analysis of genes downregulated in both transgenic conditions revealed the presence of the class 6 transporter family gene ENS_07757 (zebrafish *Slc6a4b*/mouse *Slc6a2*), whose vertebrate orthologous genes are expressed in the retina (Norton et al., 2008; Kubo et al., 2016).

No similarity in Swiss-Prot was found for the transcripts encoded by seven of the 19 DEGs dysregulated in both OC::Act and OC::Rep transgenic embryos. We further searched for functional annotation in TrEMBL, a more extensive sequence database that includes not yet curated proteins. For further investigations on the identity of uncharacterized proteins, a more general alignment was generated using the online version of the BLASTx of the transcripts vs. the nr protein database of NCBI. Then, we searched for domains using INTERPRO (https://www.ebi.ac.uk/interpro/). Neither the BLASTx- nor the INTERPRO-based analyses provided any useful information for the annotation of the considered transcripts, except for two genes, ENS_23151 and ENS_21806, downregulated in both transgenic conditions. ENS_23151 encodes a protein with a signal peptide and a non-cytoplasmic domain based on the INTERPRO analysis, indicating a possible translocation across membranes, while ENS_21806 encodes a protein carrying a Slc6sbd_SERT-like domain, the solute-binding domain of serotonin neurotransporters (data not shown) (Kristensen et al., 2011).

### Orthologous and Paralogous Oc Signaling-Dependent Genes in *Ciona* and Mouse

To identify conserved *Oc* target genes between *Ciona* and mouse *Oc* networks, we compared Oc-perturbed transcriptomes of *Ciona* PRC and mouse *Oc1/Oc*^−/−^ double knock-out retina (Sapkota et al., 2014; Supplementary Tables 1, 3). We identified 150 DEGs in the transcriptome of OC::Act and 37 in that of OC::Rep that possess a murine ortholog (Supplementary Table 1, column L). Comparison of log2 fold change (log2FC) allowed to identify eight genes (*Cacna2D3, Mak, Ebf1, Neurogenin, Gabra6, Znf385b, Prox1, Lhx5*) that were differentially expressed in both species (Sapkota et al., 2014 and Supplementary Table 3). All ascidian orthologs of the mouse DEGs were upregulated (*Znf385b, Gabra6, Cacna2d3, Prox1*) or downregulated (*Ebf1, Neurogenin, Lhx5, Mak*) only under the OC::Act transactivating form. All these genes are expressed in different territories of the vertebrate eye and encode factors involved in synaptogenesis, calcium signaling, or retinal cell specification (Supplementary Table 2). In the retina, Oc is part of a transcriptional regulatory pathway that employs *Prox1* to specify, position, and maintain horizontal cells (Wu et al., 2012, 2013; Emerson et al., 2013; Klimova et al., 2015). Accordingly, *Prox1* is significantly upregulated in *Ciona* OC::Act embryos (+2.56 log2FC). The murine ortholog of ENS_04039 *Neurogenin* gene is involved in rod PRC differentiation (*NeuroD4*) (Sapkota et al., 2014). In *Ciona, Neurogenin* is an upstream regulator of *Oc* gene (Pezzotti et al., 2014), and its downregulation in OC::Act embryos indicates a possible negative regulatory loop controlling its transcriptional activation (Supplementary Table 1). As shown by Inoue et al. (2013), members of the LIM-homeobox gene family regulate neural retina differentiation in vertebrates. Here, ascidian *Lhx5* was downregulated in OC::Act (−1.71 log2FC).

Other genes required for PRC formation in vertebrates but were not dysregulated in the mouse RNA-Seq (Sapkota et al., 2014) were differentially expressed in at least one of the two experimental conditions in *Ciona*. *Mab21* (ENS_08580) and *Opsin1* (ENS_01146) were significantly downregulated in OC::Rep condition (−1.29 and −1.52 log2FC, respectively). *Mab21* belongs to the male abnormal gene family whose members (e.g., *Mab21l1, Mab21l2*) play important roles in regulating ocular development in vertebrates (Huang et al., 2016). Among *Pax* genes, which are members of a conserved gene network that controls eye morphogenesis in numerous metazoans (Donner and Maas, 2004), the *Pax3/7* gene (ENS_13561) was significantly upregulated (+2.95 log2FC) in the OC::Act condition (Supplementary Table 1) (Ziman et al., 2001; Mazet et al., 2003; Kumar, 2009; Klimova et al., 2015).

**Figure 3:**
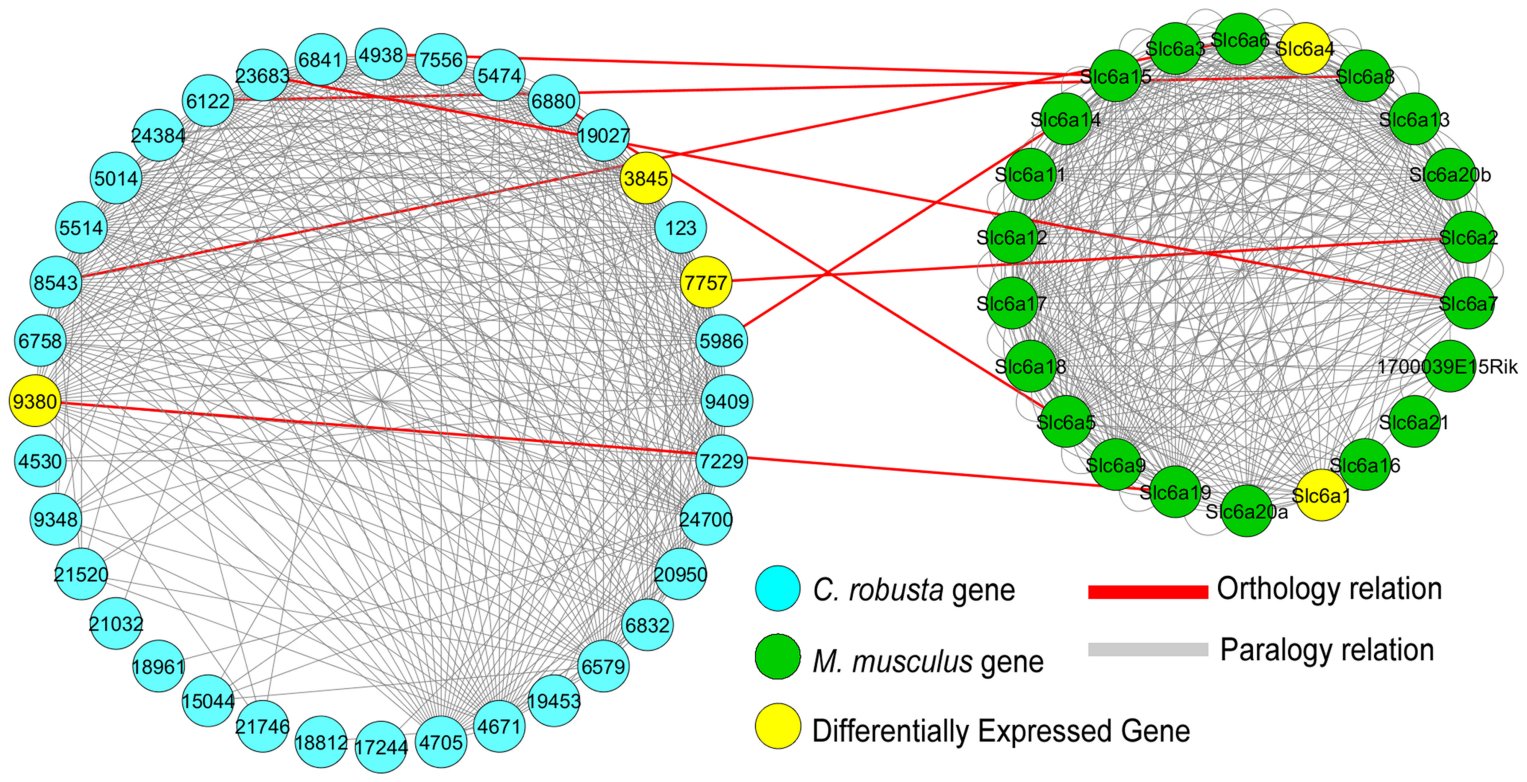
Network of *Ciona* and mouse *Slc6* paralogs and their orthology relationships. *Ciona* ENS_07757 has 35 paralogous genes (light gray edges), and among them, ENS_09380 and ENS_03845 are downregulated in the OC:Act condition (Supplementary Table 1), while mouse *Slc6a2* has 22 paralogous genes. The two paralogous gene networks are connected not only by the orthology relationship between ENS_07757 and *Slc6a2* but also by other seven relationships. Though none of the eight mouse genes that have orthology relationships with *Ciona* genes (red edges) are differentially expressed genes (DEGs) in the mouse *Oc1/Oc2*^−/−^ KO transcriptome, two alternative genes (*Slc6a1* and *Slc6a4*) among their paralogs are actually DEGs in mouse (Sapkota et al., 2014). Differentially expressed genes in both *Ciona* (ENS_03845, ENS_07757, and ENS_09380) and mouse (Slc6a4 and Slc6a1) are shown in yellow. Light blue nodes: *Ciona* Slc6 paralogs (“ENS_” in the gene names is omitted); green nodes: *M. musculus* paralogs. The visualization was obtained with Cytoscape (Shannon et al., 2003).

Considering the significant phylogenetic distance between *Ciona* and mouse, we examined the possibility that during evolution and subsequent gene duplication and divergence, a specific gene function might be conserved among paralogs of the same gene family and not necessarily between the corresponding computationally defined orthologs (Pett et al., 2019). We, hence, searched for paralogous genes of *Ciona* and mouse DEGs and analyzed their orthology relationships. Figure 3 reports the results obtained for the *Ciona* DEG *slc6a4b*/*scl6a2* (ENS_07757). This gene belongs to a group of Na^+^-dependent monoamine transporters encoded by the SLC6 gene family (Kristensen et al., 2011) that, in *Ciona savignyi*, is expanded into different subfamilies with a total of 40 putative genes (Ren et al., 2019). In our *Ciona* RNA-Seq dataset, ENS_07757 is downregulated in both OC::Rep and OC::Act conditions (Table 1), while its murine ortholog *Slc6a2* is not differentially expressed in *Oc1*/*Oc2*^−/−^ mouse (Sapkota et al., 2014). This analysis recognized the paralogy of three *Ciona* DEGs (ENS_07757, ENS_09380, and ENS_03845) with two mouse genes that belong to the same gene family of solute carrier 6 (*Slc6a1* and *Slc6a4*) and that are differentially expressed in mouse *Oc1*/*Oc2*^−/−^. Extending this analysis to all *Ciona* and mouse DEGs, we greatly increased the number of *Oc* targets shared across chordates. We found that 45 mouse DEGs are paralogs or orthologs of DEGs in *Ciona* (Supplementary Tables 3, 4). Since the relationships among paralogs are really stringent (e-value: e^−50^), we can assume that even if ENS_07757 and ENS_09380 are not the computationally defined orthologs of *Slc6a1* and *Slc6a4*, they share a high similarity level, which may be the reason for preserving the same functionality between paralogous.

### Expression of DEGs in Wild-Type and Transgenic Embryos

**Figure 4:**
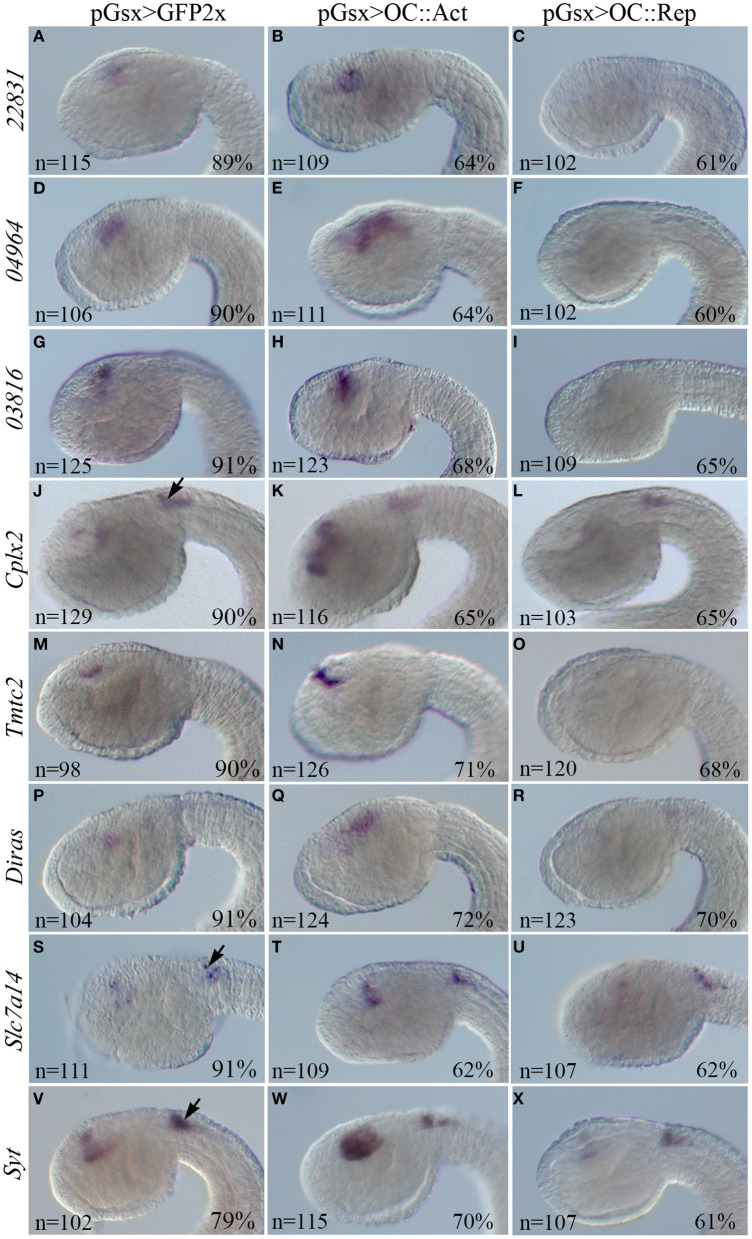
WISH in *Ciona* transgenic embryos of the DEGs inversely expressed in the OC::Act and OC::Rep conditions. WISH on transgenic embryos at tailbud 22/23 stage electroporated with the control pGsx>GFP2x **(A,D,G,J,M,P,S,V)**, the pGsx>OC::Act **(B,E,H,K,N,Q,T,W)**, or with the Gsx>OC::Rep constructs **(C,F,I,L,O,R,U,X)**. According to RNA-Seq data, all genes are upregulated in OC::Act and downregulated in OC::Rep embryos. Black arrows indicate the territory of the visceral ganglion, where no alteration of gene expression can be observed because it is outside the domain of expression of pGsx **(J–L,S–X)**. Percentages indicate the number of embryos with the corresponding expression profile in each condition as described in Supplementary Table 5. *n*, number of embryos analyzed in each condition. All embryos are in lateral view and the anterior is on the left.

To test the validity of the cell type-enriched genes identified by RNA-Seq, we looked for consistency between log2FC data and WISH results in DEGs significantly dysregulated in OC::Act and OC::Rep transgenic conditions (single WISH) and for spatial correlation between *Oc* and DEG expression patterns (double WISH) (Table 1). We generated riboprobes for 16 DEGs. Four probes (ENS_20001, ENS_23426, *Pcsk2*, and *Rab11*) showed no detectable mRNA signal in WISH, while some other riboprobes did not work in double WISH experiments. However, we show that four DEGs (*Cplx2, Tmtc2, Diras, Slc6a4b*) are specifically co-expressed with *Oc*, while one DEG (ENS_23151) is expressed in tissues adjacent to *OC*-expressing cells (Supplementary Figure 2). Importantly, eight candidate Oc targets (ENS_22831, ENS_04964, ENS_03816, *Cplx2, Tmtc2, Diras, Slc7a14, Syt*) were overexpressed in the progenitor cells of the sensory vesicle of OC::Act embryos and downregulated in OC::Rep, in agreement with log2FC in RNA-Seq datasets (Supplementary Table 1; Figures 4A–X). Expression of *Cplx2, Slc7a14*, and *Syt* genes in the visceral ganglion of transgenic embryos remained unaltered in both OC::Act and OC::Rep conditions, in support of a sensory vesicle-specific alteration (Figures 4J–L,S–X).

**Figure 5:**
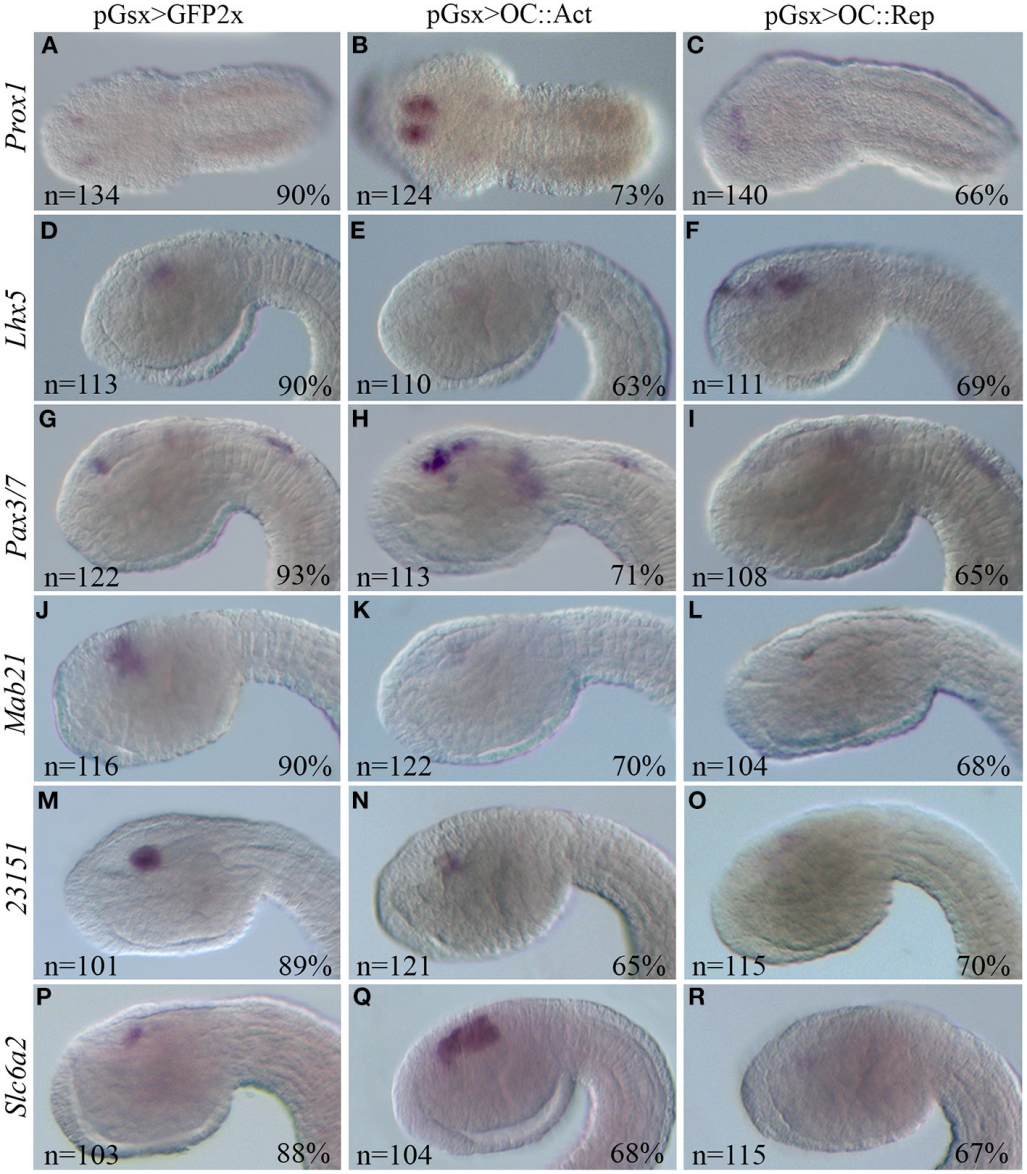
WISH of *Ciona* DEG orthologs of vertebrate retinal genes. WISH on *Ciona* embryos at tailbud 22/23 stage electroporated with the control pGsx>GFP2x **(A,D,G,J,M,P)**, with the pGsx>OC::Act **(B,E,H,K,N,Q)** or with the pGsx>OC::Rep constructs **(C,F,I,L,O,R)**. According to RNA-Seq data, *Prox1* and *Pax3*/7 are upregulated **(B,H)**, while *Lhx5* and *23151* are downregulated **(E,N)** in OC::Act embryos; *Mab21, 23151*, and *Slc6a2* are downregulated in OC::Rep embryos **(L,O,R)**. The only exception to RNA-Seq data is upregulation of *Lhx5* in OC::Rep **(F)** and *Slc6a2* in OC::Act **(Q)** embryos. Percentages indicate the number of embryos with the corresponding expression profile in each condition as described in Supplementary Table 5. All embryos are in lateral view, except for **(A–C)** in dorsal view. The anterior is on the left.

Concerning genes that are similarly dysregulated in both RNA-Seq datasets or only in one, we selected several genes required for proper vertebrate retinal tissue formation. In particular, we identified two genes, *Prox1* and *Lhx5*, which are dysregulated in our RNA-Seq dataset and in that of mouse *Oc1/Oc2*^−/−^ double mutant retinas (Sapkota et al., 2014 and Supplementary Table 3). These genes are upregulated and downregulated in the OC::Act dataset, respectively; accordingly, they show increased (*Prox1*) and reduced (*Lhx5*) transcript staining in the sensory vesicle of OC::Act transgenic tailbud stage 22 embryos in comparison with controls (Figures 5A–F). The only exception is *Lhx5* upregulation in OC::Rep embryos that is not supported by RNA-Seq data (Figure 5F). We further analyzed the expression of *Ciona Pax3/7* (ENS_13561) and *Mab21* (ENS_08580), orthologous genes of two important factors for PRC formation (Klimova et al., 2015; Huang et al., 2016). In agreement with RNA-Seq data, *Pax3/7* was overexpressed in OC::Act and *Mab21* was downregulated in OC::Rep tadpole embryos (Supplementary Table 1; Figures 5G–L).

WISH results were consistent also for the uncharacterized ENS_23151 gene, which shows reduced mRNA labeling in the sensory vesicle of OC::Act embryos as well as no detectable expression in OC::Rep embryos (Table 1; Figures 5M–O). Conversely, *Slc6a2* (ENS_07757), which shows a negative log2FC in both OC::Act and OC::Rep RNA-Seq datasets, is downregulated only in the sensory vesicle of OC::Rep embryos and is upregulated in OC::Act embryos (Table 1; Figures 5P–R). Whereas, WISH results reveal a considerable level of reliability of the two RNA-Seq datasets, they prompt the necessity to functionally validate the results obtained in order to provide a correct pathway analysis.

## Discussion

A transgenic approach was used to identify downstream target genes of the Oc transcription factor in ascidian PRC. Comparative analysis of the data from both constitutively active and constitutively repressive forms of Oc showed significant dysregulation of genes involved in different traits of synaptic activity, such as exocytosis, calcium transport, and neurotransmission (Table 1; Supplementary Table 2). Furthermore, components of the *Oc* genetic pathway conserved between the distantly related species *Ciona* and mouse provide a framework for understanding how PRC subtype diversification originated during evolution.

### Mechanisms by Which *Oc* Functions in Ascidian Photoreceptors

**Figure 6:**
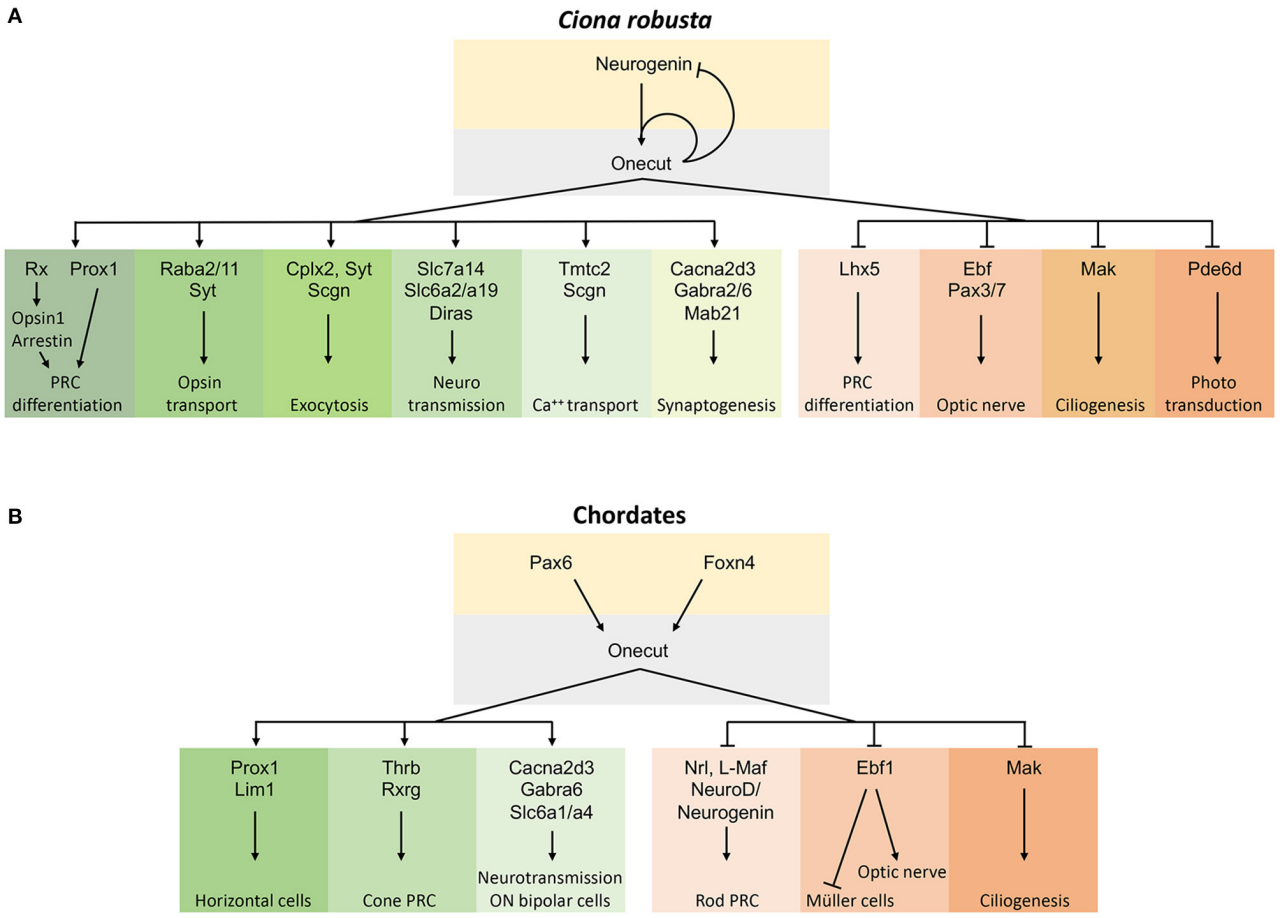
Schematic representations of the gene regulatory pathway controlled by Oc factors in the chordate retina. **(A)** Oc gene network in *Ciona* PRC differentiation where only genes with a corresponding vertebrate ortholog/paralog are reported. **(B)** Oc gene network in chordate retina obtained by comparing the *Ciona* and vertebrate Oc target genes. Arrows within the gene network solely represent our current understanding of network relationships and do not imply genetic mechanisms such as direct or indirect transcriptional regulation. Oc-activated targets are on the left, repressed ones on the right.

Using transgenesis, RNA-Seq, bioinformatics, and WISH, we provide additional insights into which gene transcripts are implicated in the Oc genetic cascade in *Ciona* PRC (D'Aniello et al., 2011; Pezzotti et al., 2014). Our findings suggest novel Oc-dependent mechanisms underlying the formation of the PRC type in ascidians that had not been linked to Oc signaling previously. The results of this study show a robust relationship and interaction with 14 DEGs that were inversely expressed between the two transgenic conditions. Several of these genes are key components in fundamental systems of information processing like exocytocis (*Cplx2, Syt, Scgn*), neurotransmission (*Slc7a14, Diras*), opsin transport (*Rab11, Syt*), and calcium homeostasis (*Tmtc2, Scgn*). Given the activating or inhibitory role of Oc on gene expression, we propose that *Ciona* Oc serves as a regulator of proteins that mediate synaptic activities. Furthermore, *Oc* itself and some of these DEG genes with neurosynaptic activity are not restricted to PRC, suggesting that Oc could use the same target genes to promote neural cell type differentiation in various regions of the nervous system (Figure 6A). Chip-Seq data will be needed to clarify if the specific role of Oc in the regulation of these DEGs is direct or indirect.

### Conserved *Onecut* Genetic Cascade in Developing Photoreceptors in the Phylum Chordata

Comparative examination of Oc downstream targets in ascidian and mouse transcriptomes could provide hints on the evolution of the Oc regulatory network. At the rise of vertebrates, Oc genetic cascade has acquired additional functions in a wider range of retinal cell types, including glial and interneuron cell specification (*Cacna2d3, Ebf1*), PRC differentiation (*Neurogenin, Lhx5, Prox1*), inhibition of Müller cell differentiation and positive regulation of optic nerve projections (*Ebf1*), ciliogenesis (*Mak*), synapsis formation, and GABA release (*Cacna2d3, Gabra6*) (Kurshan et al., 2009; Nakajima et al., 2009; Jin et al., 2010; Jin and Xiang, 2011; Kourakis et al., 2019). Altogether, observations obtained in *Ciona* and mouse indicate that Oc factors regulate the expression of a common set of orthologous and possibly paralogous genes during development to promote or inhibit differentiation of different cell types and functions in the chordate eyes (Klimova et al., 2015) (Figure 6B).

In mouse, *Oc1*^−/−^*/Oc2*^−/−^ double knock-out revealed a fundamental role played by these transcription factors in the formation of HC, RGC, and amacrine cell types, but not of PRC (Goetz et al., 2014; Sapkota et al., 2014; Klimova et al., 2015). However, a dominant-negative form of mouse *Oc1* does not affect the total number of PRC but promotes the differentiation of rods at the expense of cones (Emerson et al., 2013; Jean-Charles et al., 2018). Mouse Oc proteins control rod vs. cone PRC differentiation by negatively regulating the Nrl transcription factor, a rhodopsin activator (Mears et al., 2001). The expression of the ascidian ortholog of the vertebrate *Rhodopsin* gene, *Opsin1* (ENS_01146), and of the rhodopsin-transporter *Rab11a* (ENS_02864) decreased under a predominantly repressive Oc regulation (OC::Rep) (Kusakabe et al., 2001). Of note, the expression of *Pde6d* (ENS_3961), the gene coding for the delta subunit of rod-specific photoreceptor phosphodiesterase (Pde6d), a key enzyme in the phototransduction cascade, dropped under the transactivating condition (Supplementary Table 1). These results might be consistent with Oc implication in visual opsin synthesis in ascidian PRC and with *Ciona* Oc activating a rod-specific repressive mechanism, suggesting that rod cells evolved from cone cells in the common ancestors of mammals and fish (reviewed in Asteriti et al., 2015). Therefore, our results could provide important information to unveiling the functional properties of cone PRC and to understand the genetic mechanisms of cell subtype diversification in retina evolution. Further studies on other vertebrate models will be necessary to confirm the mammalian-specific role of *Oc* genes in cone differentiation and to determine the precise downstream synaptic signaling mechanisms resulting from the Oc genetic cascade across chordate evolution (Figure 6).

Finally, our bioinformatics analysis suggests how specific gene functions may be shared by paralogous genes and not by computationally defined orthologous genes. This may be the case of *Slc6* and *Lim* gene families, with *Slc6a2, Slc6a19*, and *Lhx5* DEGs in *Ciona* and their paralogs *Slc6a1, Slc6a4, Lhx4*, and *Lhx9* DEGs in mouse (Sapkota et al., 2014; Figure 6). This underlines the importance of further considering paralogy relationships in the definition of the roles of specific genes in regulatory pathways and paves the way to deeper investigations to estimate the levels of genetic conservation in their evolutionary story and among species (Pett et al., 2019).

## Conclusions

In this study, we contribute in understanding the cascade of events that direct the formation of the increasingly complex eye structures of chordates. It was probably the close functional and regulatory interaction among *Oc* members that contributed to keep hidden their role in promoting PRC differentiation. Full comprehension of *Oc* role in photoreceptor differentiation could represent an important turning point to understand the process that contributed to cone and rod diversification in vertebrates. These findings led us to propose that the small-eye phenotype observed in ascidian transgenic larvae and mouse *Oc1*/*Oc2*^−/−^ knock-outs reflects defects in various aspects of cell differentiation, division, maintenance, and physiology, thus indicating a broad role of Oc proteins in PRC development. Remarkably, several new genes of the *Oc* network have human orthologs that are associated with retinal pathologies, such as retinitis pigmentosa, glaucoma, retina ciliopathy, diabetic retinopathy, and macular degeneration. With the evolutionary diversification of retinal neurons and interneurons, *Oc* genes could have been used to control cell signaling in the process of light impulse transduction in the Chordata phylum.

## Data Availability Statement

The datasets presented in this study can be found in online repositories. The names of the repository/repositories and accession number(s) can be found at: https://www.ncbi.nlm.nih.gov, PRJNA680353.

## Author Contributions

QV, AF, GF, VN, and FB did the molecular experiments. CC did the bioinformatics analyses. QV, CC, AL, and PS drafted the manuscript and prepared the figures. RK, AL, and PS supervised the experiments. MC supervised the bioinformatics analyses. PS and AL edited and revised the manuscript. All authors approved the manuscript for publication.

## Conflict of Interest

The authors declare that the research was conducted in the absence of any commercial or financial relationships that could be construed as a potential conflict of interest.

## Supplementary Material

Raw images collection: http://dev.aniseed.cnrs.fr/aniseed/experiment/list_insitus_by_pub?pub_id=321.
