# Supplementary material for: Onecut Regulates Core Components of the Molecular Machinery for Neurotransmission in Photoreceptor Differentiation
Source: Front Cell Dev Biol. 2021 Mar 18;9:602450. doi: 10.3389/fcell.2021.602450 (PMC8012850; doi:10.3389/fcell.2021.602450)
Supplement: Supplementary file 2 [file Table_2.docx]

**Supplementary Table 2. Genes differentially expressed in OC::Act and/or OC::Rep embryos**

**involved in vertebrate retinal tissues formation, synaptogenesis and neurotransmission.**

| **Ensembl code** | **Encoded Protein** | **Name** | **Function**  **in vertebrate eye** | **Referencee** |
| --- | --- | --- | --- | --- |
| ENS_05712 | Subtilisin/kexin-type 2 | *Pcsk2* | proliferative retinopathy | Leak et al., 2007; Grassi et al., 2011; Graham et al., 2018 |
| ENS_05701 | Secretagogin-like | *Scgn-like* | exocytosis, calcium homeostasis | Puthussery et al., 2010; Qin et al., 2020 |
| ENS_02864 | Small GTPase | *Rab11a* | rhodopsin transport | Ying et al., 2016 |
| ENS_20001 | FK506-binding protein 3 | *FKBP25/3* | neurotransmission | Kitagawa et al., 1996 |
| ENS_13789 | Calcium channel alpha2/delta subunit 3 | *Cacna2d3* | synaptogenesis | Kurshan et al., 2009; |
| ENS_04249 | Gamma-aminobutyric acid receptor subunit alpha-6 | *Gabra6* | GABA release | Zega et al., 2008; Nakajima et al., 2009; Monesson-Olson et al., 2018 |
| ENS_7420 | zinc finger protein | *Znf385b* | microphthalmia, neurodegeneration | Wu et al., 2002; Mencarelli et al., 2007; Han et al., 2020 |
| ENS_17973 | NA | *Mak* | cilium-associated | Stone et al., 2011; van Huet et al., 2015; Gray et al., 2018 |
| ENS_03209 | early B-cell factor helix-loop-helix transcription factor | *Ebf1* | differentiation, axogenesis | Jin et al., 2010; Jin and Xiang, 2011; Chuang et al., 2018 |
