# Supplementary material for: Onecut Regulates Core Components of the Molecular Machinery for Neurotransmission in Photoreceptor Differentiation
Source: Front Cell Dev Biol. 2021 Mar 18;9:602450. doi: 10.3389/fcell.2021.602450 (PMC8012850; doi:10.3389/fcell.2021.602450)
Supplement: Supplementary file 6 [file Table_6.docx]

**Supplementary Table 6. Primers used for PCR amplifications**

| ***Ciona* gene name** | **Oligo Fw** | **Oligo Rev** | **Probe length** |
| --- | --- | --- | --- |
| *23151* | 5’-CTGCTTAACCATAATGTCGAGTAC-3’ | 5’-GAATTTACAACACGACGACGTC-3’ | 739 bp |
| *Tmtc2* | 5’-CTTGTCTCGATGATTCTGCGAC-3’ | 5’-AATGTGACGGCACGTGATCTGG-3’ | 1354 bp |
| *Diras* | 5’-GAACATGGTGATATACTGGTTCG-3’ | 5’-CTAATCGTTCCACGGTTGACTAG-3’ | 864 bp |
| *Slc6a4b* | 5’-GAATGGCAGACAACTGCTATGC-3’ | 5’-GAGTGACGTAAAGTACATTGACC-3’ | 1219 bp |
| *04964* | 5’-TTCGCCGGATCAAATCAATAAGC-3’ | 5’-TAACACTGAGTTCTTGGCGAAAG-3’ | 979 bp |
| *03816* | 5’-AGGAACTTGCATCGACGTGTGG-3’ | 5’-GAATGTACAGAGTCGTGTGTACG-3’ | 1133 bp |
| *Slc7a14* | 5’-AGGAGCGAGTGCAATAAGTAGC-3’ | 5’-GCCATGTATGCCAATCTTGTAGG-3’ | 1257 bp |
| *Mab21* | 5’-CTACAACGAGCAAACGGTGC-3’ | 5’-TTGGTCCATTCCTCGTCACG-3’ | 834 bp |
| *Proxa* | 5’-GTCACTTGCAACACGCAGCTTCC-3’ | 5’-GCGTCACAGAGTCGATCGAGAAC-3’ | 1192 bp |
| *Lhx5* | 5’-CTCGTCGAATCGTGCTTCAC-3’ | 5’-AACTGTCACGAGGTGATGTC-3’ | 1144 bp |
| *Cplx2* | 5’-TCATAGTGAAGCAAGCACTCGG-3’ | 5’-TCGACTATATAACACTCGCACACG-3’ | 974 bp |
| *22831* | 5’-GAACGATTCAGTTCATTTGAATGAC-3’ | 5’-CCACATATTTGACTCAAGTCATCG-3’ | 1151 bp |
