# Supplementary figures and images for: Onecut Regulates Core Components of the Molecular Machinery for Neurotransmission in Photoreceptor Differentiation
Source: Front Cell Dev Biol. 2021 Mar 18;9:602450. doi: 10.3389/fcell.2021.602450 (PMC8012850; doi:10.3389/fcell.2021.602450)

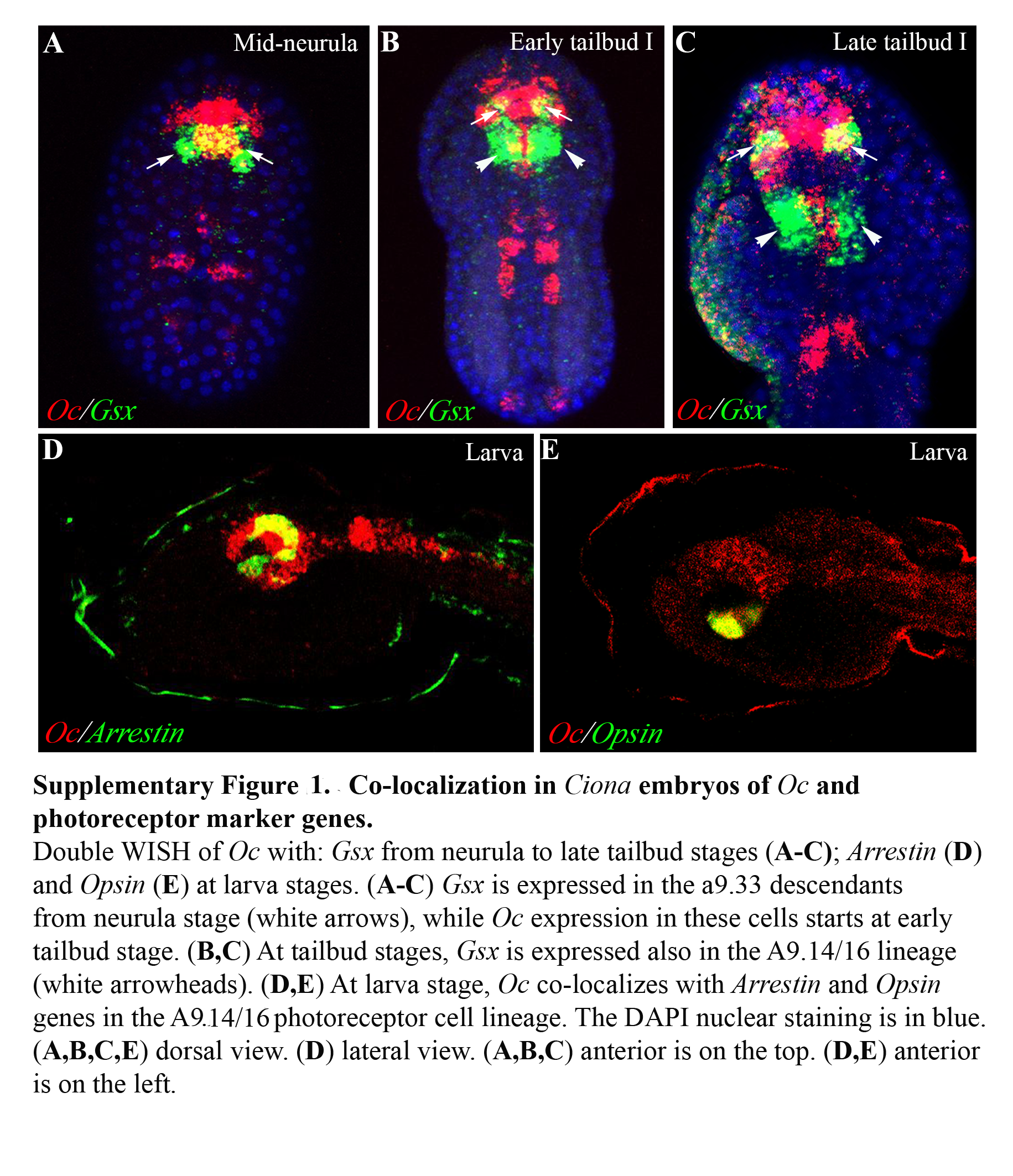

Supplement: Supplementary file 7 [file Image_1.tif]

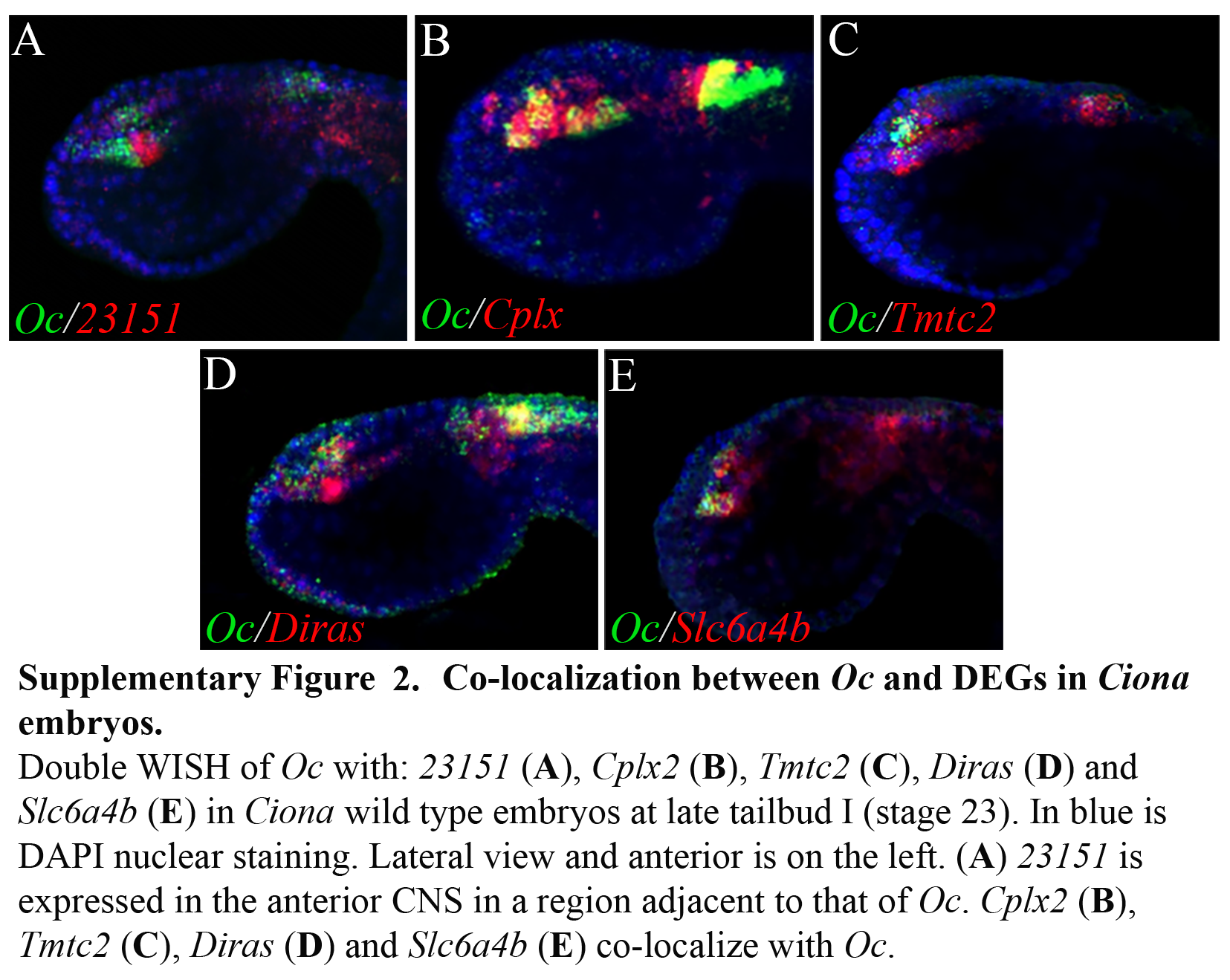

Supplement: Supplementary file 8 [file Image_2.tif]
